# Examination of the autonomic nervous system in the ICU: a pilot study

**DOI:** 10.1186/cc10904

**Published:** 2012-03-20

**Authors:** L Wieske, E Kiszer, C Verhamme, IN Van Schaik, MJ Schultz, J Horn

**Affiliations:** 1 Academic Medical Center, Amsterdam, the Netherlands

## Introduction

The most widely used test for autonomic dysfunction in the ICU is the heart rate variability (HRV) test [1]. HRV is thought to be a very sensitive but less specific test [1]. Several other tests are available. For this pilot study we have investigated the ability of two tests, the skin wrinkle test (SWT), a test for postganglionic sympathetic function, and the cold face test (CFT), a reflex slowing heart rate after cold application to the forehead, to detect autonomic dysfunction in critically ill patients alongside the HRV.

## Methods

ICU patients mechanically ventilated for at least 3 days were included. Exclusion criteria: polynomic or autonomic neuropathy, admission after stroke or cardiac arrest. HRV was investigated using power spectral analysis of continuous 5-minute ECG recordings [1]. The simulated SWT was used and wrinkling was assessed on a five-point scale [2]. Under continuous ECG recording a cold pack was applied to measure the CFT [3]. Changes in SWT and CFT results over time were compared to the changes in the SOFA score. Studies procedures were also performed in 17 healthy controls.

## Results

**Figure 1:**
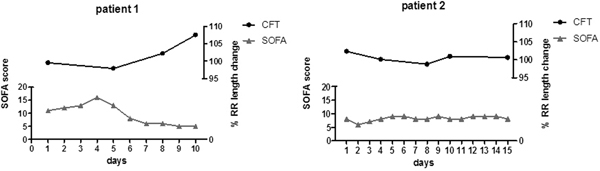
**Changes in cold face test (CFT) results over time**.

Twelve patients were included (mean age: 54 (SD: 15)). HRV analysis showed decreased heart rate variability in all patients (median total power: 32 ms^2 ^(IQR: 11 to 320)). The SWT could be performed in 10 patients. SWT results were abnormal (score ≤2) in 60% of cases (6% in healthy controls; *P *< 0.01). The CFT was done in nine patients. Critically ill patients showed a blunted response on the CFT (2.5% increase in RR length (95% CI: -0.2% to 5.2%) vs. 7.1% in healthy controls (95% CI: 3.7% to 10.5%; *P *= 0.03)). Figure 1 displays the CFT results over time.

## Conclusion

CFT detected autonomic dysfunction in critically ill patients better than the SWT and was easier to perform. Diagnostic accuracy and prognostic value need to be investigated.
